# A simulation study of differentially private federated learning with zero-trust policy screening for medical IoT devices

**DOI:** 10.3389/fdgth.2026.1807342

**Published:** 2026-09-24

**Authors:** Ihssan S. Masad, Ali Mohammad Alqudah, Shoroq Qazan, Sultan Shoaib

**Affiliations:** 1 Department of Electrical and Computer Engineering, Gulf University for Science and Technology (GUST), Hawally, Kuwait; 2 Department of Biomedical Systems and Informatics Engineering, Yarmouk University, Irbid, Jordan; 3 Lucidya Tech LTD, Winnipeg, MB, Canada

**Keywords:** device trust scoring, federated learning, Gaussian differential-Privacy mechanism, gradient-Magnitude anomaly screening, internet of medical things (IoMT), privacy-preserving machine learning, zero-Trust policy engine

## Abstract

Artificial intelligence is increasingly used in connected medical-device systems for physiological-signal classification and continuous monitoring. This paper presents a simulation study of the learning and policy layers of a zero-trust federated-learning framework for Internet of Medical Things (IoMT) devices. The implemented prototype combines federated averaging, Gaussian perturbation of client updates, a device-level trust score, and gradient-magnitude screening of anomalous client updates. This is a simulation study of the federated-learning and zero-trust policy layers; cryptographic, encrypted-transport, and hardware-attestation components are modeled as deployment considerations or overheads and are not implemented as complete physical or protocol-level modules. Using the ECG classification pipeline described in the methodology, we compare centralized training, traditional federated learning, differentially private federated learning, and the proposed zero-trust federated configuration. The proposed ZT-FL configuration is reported at 0.928 accuracy, 0.920 F1-score, and 0.968 AUC. These results support feasibility within the stated simulation scope and do not establish clinical validity, formal end-to-end DP accounting, comprehensive adversarial robustness, regulatory compliance, or post-quantum readiness.

## 1 Introduction

The integration of artificial intelligence into medical devices has revolutionized healthcare delivery, enabling real-time patient monitoring, early disease detection and personalized treatment recommendations (1). Medical Internet-of-Things (IoMT) devices including wearable sensors, implantable devices and connected diagnostic equipment generate vast amounts of sensitive physiological data that can be leveraged to train sophisticated machine learning models (2). These models have demonstrated remarkable performance in detecting cardiac arrhythmias, predicting respiratory failures and identifying early signs of chronic diseases (3). However, the increasing connectivity and computational sophistication of medical devices have introduced critical security and privacy vulnerabilities (4). Traditional centralized machine learning approaches require the aggregation of sensitive patient data in a central repository, raising significant concerns regarding data breaches, unauthorized access and regulatory non-compliance with standards such as HIPAA and GDPR (5). Furthermore, centralized systems create single points of failure and may not be feasible in resource-constrained IoMT environments where devices operate with limited computational power and battery capacity (6). Federated Learning (FL) has emerged as a promising paradigm to address these challenges by enabling collaborative model training without centralizing sensitive data (7). In FL, individual devices retain their data locally and only share model updates (gradients) with a central server, which aggregates these updates to produce a global model (8). This approach significantly reduces the risk of data exposure while enabling distributed intelligence across IoMT networks (9). Despite its advantages, traditional Federated Learning systems assume partial trust between clients and servers (10). This assumption is inadequate for the high-stakes, safety-critical environment of medical devices, where even a single compromised device or malicious server could lead to catastrophic consequences (11). Recent research has demonstrated that FL systems are vulnerable to multiple attack vectors including gradient inversion attacks that can reconstruct sensitive training data from shared model updates (12). Model inversion attacks that extract information about individual patients (13) and adversarial attacks that manipulate model predictions can cause incorrect diagnoses (14, 15).

To address these challenges, a novel Zero-Trust Federated Learning (ZT-FL) framework is proposed that fundamentally reimagines the trust model in distributed medical AI systems. The ZT-FL framework integrates three core components: (1) a Zero-Trust Policy Engine (ZTPE) that enforces continuous verification and authorization of all devices and communications; (2) an Adaptive Differential Privacy (A-DP) mechanism that adjusts privacy budgets based on device computational capacity, battery status and data sensitivity; and (3) a Secure Aggregation protocol that prevents intermediate servers from observing individual model updates. The principal novelty of the proposed framework lies in integrating a lightweight 1D-CNN local classification model within a secure and adaptive federated-intelligence framework for consumer IoMT environments. Unlike conventional deep learning or standard federated learning approaches that mainly focus on predictive accuracy, the proposed ZT-FL framework combines continuous Zero-Trust verification, device-level trust scoring, adaptive differential privacy, secure aggregation, encrypted mobile communication, and anomaly-based model-update validation within a unified healthcare-AI pipeline. This integration enables the framework to preserve patient privacy, reduce the risk of model inversion and gradient reconstruction attacks, and maintain reliable predictive performance under heterogeneous and resource-constrained consumer medical-device conditions. Thus, the main contribution of this work is the integrated trustworthy federated-learning framework rather than the standalone CNN architecture. The main contributions of this research work are:

- Trustworthiness Framework for Consumer IoMT: ZT-FL integrates Zero-Trust policies (identity, posture, behavior) with privacy-preserving FL to harden consumer medical wearables.
- Proactive Risk Detection: Predictive threat modeling detects poisoning and exfiltration before aggregation using gradient-magnitude drift and attestation failures.
- AI-Guided Security Validation: Anomaly-based screening of model updates is incorporated into the simulated ZTPE to identify suspicious or potentially malicious client behavior. More extensive AI-guided validation using auxiliary or shadow models is retained as an architectural extension for future deployment.
- Integrity & Encrypted Mobile Channels: mTLS, PFS, signed updates and secure aggregation ensure mobile integrity and confidentiality.
- Post-Quantum and Authentication Deployment Readiness: The framework identifies FIDO2/TPM-based authentication and post-quantum KEM/TLS migration as deployment-oriented security extensions. These mechanisms are discussed as architectural pathways for future consumer-IoMT implementation and were not directly implemented as hardware or post-quantum cryptographic modules in the present simulation.

This paper is organized as follows: Section 2 reviews related work on Federated Learning, Zero-Trust Architecture and Differential Privacy. Section 3 presents the detailed architecture of the ZT-FL framework. Section 4 describes the experimental methodology. Section 5 presents and analyzes the experimental results including cost analysis. Section 6 concludes the paper and outlines future work followed by acknowledgements.

## 2 Related work

The rapid digital transformation of healthcare has accelerated the adoption of artificial intelligence (AI) and machine learning (ML) for clinical decision support, disease diagnosis and personalized medicine. However, the highly sensitive nature of medical data and stringent privacy regulations, such as the Health Insurance Portability and Accountability Act (HIPAA) and the General Data Protection Regulation (GDPR), pose significant barriers to centralized model training (16). To address these challenges, Federated Learning (FL) has emerged as a promising standard that enables collaborative model development across distributed healthcare institutions without sharing raw patient data. However, the deployment of FL in healthcare raises new concerns regarding data heterogeneity, communication overhead, and cybersecurity vulnerabilities within interconnected medical ecosystems (17). Integrating Zero-Trust Architecture (ZTA) and privacy-preserving techniques such as Differential Privacy (DP) and Secure Aggregation (SecAgg) provides a robust framework to enhance security, trust and compliance in federated healthcare systems (18). This review explores the intersection of these emerging technologies, FL, ZTA and privacy-preserving mechanisms and highlights their current applications, challenges and future directions for building secure, trustworthy and scalable intelligent healthcare systems. While the focus is medical AI, the device class is predominantly consumer electronics (smartwatches, fitness bands, home ECG patches and smartphone-paired sensors). These platforms face consumer-app threat models from jailbroken phones, app-store side-loads, insecure home routers and shared family devices. Accordingly, ZT-FL is positioned to anticipate, identify and counter consumer-space threats through predictive threat modeling, continuous verification and privacy-preserving local learning.

### 2.1 Federated learning in healthcare

Federated Learning has emerged as a transformative method for collaborative machine learning in healthcare to address the critical challenge of training models on sensitive patient data without centralizing that data (19). Recent systematic reviews highlight the widespread adoption of FL across diverse medical applications, including medical imaging analysis, disease prediction and clinical decision support systems (20, 21). The integration of FL with Internet of Things (IoT) devices and wearables has opened new possibilities for real-time patient monitoring and personalized healthcare delivery (22). Smart health systems leverage FL to enable continuous learning from distributed medical devices while preserving patient privacy (23). Applications range from arrhythmia detection in wearable ECG monitors to respiratory failure prediction in intensive care units (24). However, existing FL implementations in healthcare face significant challenges. Data heterogeneity (non-IID distribution) across different medical institutions and device types can degrade model performance (25). Communication overhead in bandwidth-constrained IoMT networks can make FL impractical (26). Additionally, the governance and regulatory compliance of FL systems in healthcare remains an open research question (27).

### 2.2 Zero-Trust architecture (ZTA)

Zero-Trust Architecture represents a paradigm shift in cybersecurity, moving away from the traditional perimeter-based security model toward a principle of “never trust, always verify” (28). In ZTA no user, device or application is inherently trustworthy regardless of whether it is inside or outside the network boundary (29). Every access request must be authenticated, authorized and continuously monitored (30). ZTA has gained significant traction in healthcare cybersecurity where protecting sensitive patient data is paramount (31). The application of ZTA to Internet of Medical Things (IoMT) devices presents a natural fit as medical devices often operate in heterogeneous environments with varying security postures (32). By applying equal skepticism to all devices, Zero-Trust Architecture (ZTA) helps prevent attackers from moving laterally within the network and minimizes the potential damage caused by a compromised device (33).

### 2.3 Privacy-Preserving techniques in federated learning

Differential Privacy (DP) has become the gold standard for privacy protection within the stated simulation scope in machine learning (34). DP provides a mathematically rigorous framework for quantifying privacy loss through the privacy budget parameter *ε*, where smaller *ε* values correspond to stronger privacy guarantees (35). In FL, DP is typically implemented by adding calibrated noise to local model updates before transmission to the server (36). Recent advances in adaptive differential privacy have demonstrated the benefits of dynamically adjusting privacy parameters based on data characteristics and model properties (37). A-DP mechanisms can improve model utility by injecting less noise for less sensitive features while maintaining strong privacy guarantees for sensitive information (38). SecAgg is another critical privacy-preserving technique in FL, enabling the central server to compute the aggregate of client model updates without observing individual updates (39). SecAgg protocols typically employ cryptographic techniques such as homomorphic encryption or secure multi-party computation (40).

### 2.4 Security challenges in IoMT

The increasing connectivity and sophistication of IoMT devices have introduced multiple security vulnerabilities (37). Model inversion attacks allow adversaries to reconstruct sensitive training data from trained models (33). Gradient inversion attacks exploit shared model updates in FL to recover individual training samples with high fidelity (46). Data reconstruction attacks can extract sensitive patient information from gradient updates (47). Adversarial attacks pose another significant threat, where carefully crafted perturbations to input data can cause misclassification in deep learning models (48). In the medical domain, adversarial attacks could lead to incorrect diagnoses or treatment recommendations with potentially fatal consequences (48).

### 2.5 Recent advances in explainable, edge and federated intelligence

Recent studies in consumer electronics, edge intelligence and trustworthy AI highlight the need for secure, explainable and resource-aware learning frameworks. For example, SFX-GAN demonstrates the importance of sustainable and explainable multi-modal spectral fusion in complex imaging systems. It shows how interpretability and computational efficiency can be integrated into deep learning pipelines (41). Similarly, adaptive edge intelligence frameworks for resource-constrained IoT devices emphasize the importance of lightweight learning and decision-making under limited computational and communication resources (42). In healthcare-oriented intelligent systems, neuro-symbolic digital twins have been explored for cardiovascular disease prediction and personalized modelling which demonstrates the growing role of hybrid AI models in clinically relevant predictions (43). Optimized federated learning for trustworthy edge decision-making in IoT consumer electronics has shown that federated intelligence must be strengthened with trust-aware mechanisms to support reliable deployment in distributed environments (44). Recent optimization-based federated learning methods such as dynamic inertia weight-based advanced particle swarm optimization also indicate that adaptive optimization can improve federated learning performance on consumer electronic devices (45). In comparison with these studies, the proposed ZT-FL framework specifically integrates Zero-Trust verification, A-DP, SecAgg and IoMT-focused deployment constraints into a unified framework for privacy-preserving and trustworthy healthcare intelligence.

## 3 Proposed zero-trust federated learning framework

As healthcare systems increasingly depend on interconnected Internet of Medical Things (IoMT) devices and distributed machine learning, maintaining data security, privacy and trust throughout the network has emerged as a critical challenge. Traditional federated learning (FL) architectures, while effective in preserving data locality, remain vulnerable to adversarial attacks, data leakage and compromised devices within the training network. To overcome these limitations, the Zero-Trust Federated Learning (ZT-FL) framework integrates Zero-Trust security principles, adaptive differential privacy (A-DP) and secure aggregation mechanisms into a unified architecture. This framework enforces continuous verification of devices, dynamic privacy adaptation based on device constraints and end-to-end encrypted model communication. By combining these layers of security and privacy, ZT-FL establishes a resilient, privacy-aware and trustworthy federated learning environment suitable for sensitive healthcare applications. The following sections describe the architecture and components of the ZT-FL framework, including the Zero-Trust Policy Engine (ZTPE), Adaptive Differential Privacy mechanism and Secure Aggregation protocol which together ensure secure, compliant and efficient collaborative learning in IoMT ecosystems.

### 3.1 Architecture overview

Scope and implementation boundary. This work evaluates the federated-learning, Gaussian update-perturbation, and zero-trust policy layers in simulation. Complete secure aggregation, encrypted transport, mutual TLS, TPM/FIDO2 attestation, and post-quantum mechanisms are modeled as deployment considerations or overheads rather than implemented cryptographic or hardware modules.

The ZT-FL framework consists of three main components: (1) a distributed network of IoMT client devices, (2) a central aggregation server and (3) a Zero-Trust Policy Engine (ZTPE) that governs all interactions between clients and the server. Figure 1 illustrates the high-level architecture of the ZT-FL system showing the interaction between IoMT devices, local training with A-DP, secure aggregation and the Zero-Trust Policy Engine. The high-level workflow of ZT-FL is as follows:

- Device Registration and Verification: Each IoMT device registers with the ZTPE and undergoes strong identity verification using hardware-backed credentials (e.g., TPM-based attestation).
- Local Training with A-DP: Devices perform local model training on their private data. Before sharing model updates, devices apply adaptive differential privacy noise injection based on their computational and energy constraints.
- Secure Aggregation: The central server receives encrypted model updates from devices and performs secure aggregation without observing individual updates.
- Continuous Verification: Throughout the process, ZTPE continuously monitors device behavior, network traffic and model updates for anomalies or signs of compromise.
- Global Model Update: The aggregated model is securely distributed back to devices for the next training round.

**Figure 1:**
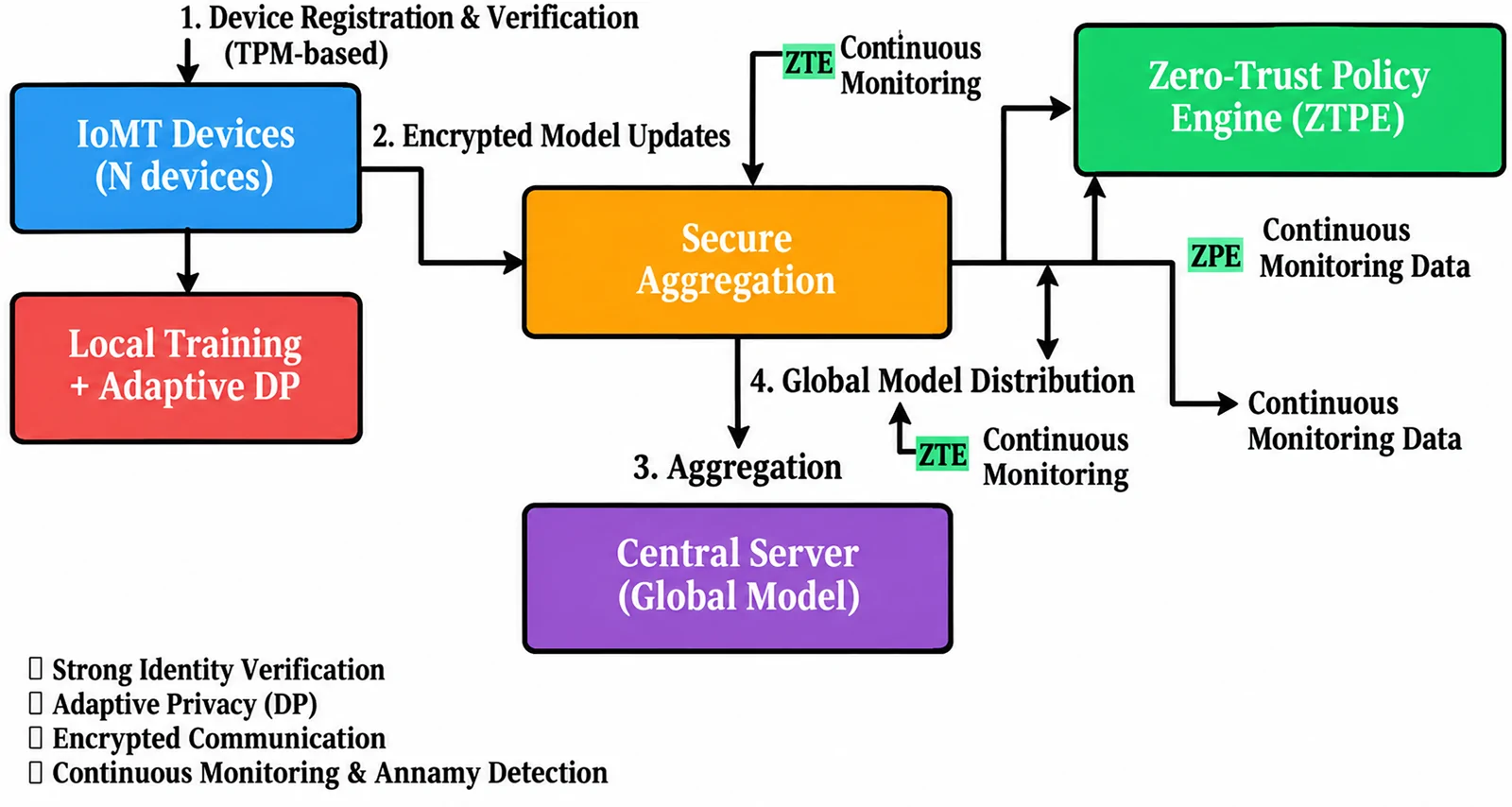
Zero-Trust federated learning system architecture.

### 3.2 Zero-Trust policy engine (ZTPE)

The prototype uses fixed policy parameters of 0.70 for exclusion, 0.85 for restriction, and 3× the mean gradient magnitude for anomaly screening. These are proof-of-concept operating points, not learned universal optima; threshold sensitivity remains a limitation.

The ZTPE is the core component that enforces Zero-Trust principles in the ZT-FL framework. It maintains a dynamic trust score for each device based on multiple factors:

- Device Identity Verification: Each device must possess a unique, hardware-backed credential (e.g., from a Trusted Platform Module) that cannot be spoofed or transferred. The ZTPE verifies this credential at the start of each training round and at regular intervals during the round.
- Device Health Assessment: ZTPE monitors the security posture of each device including the presence of security patches, antivirus software and absence of known vulnerabilities. Devices with degraded security postures receive lower trust scores and may be excluded from critical training rounds.
- Behavioral Anomaly Detection: The ZTPE analyzes the behavior of each device during training including the magnitude of model updates, the frequency of communications and the consistency of updates across rounds. Devices exhibiting anomalous behavior are flagged for investigation. Figure 2 shows the distribution of device trust scores in a typical ZT-FL deployment.
- Contextual Access Control: Access to the global model and participation in training rounds is determined by the device's current trust score, the sensitivity of the data being trained and the criticality of the model. Table 1 presents sample policy rules for the ZT-FL framework.

**Figure 2:**
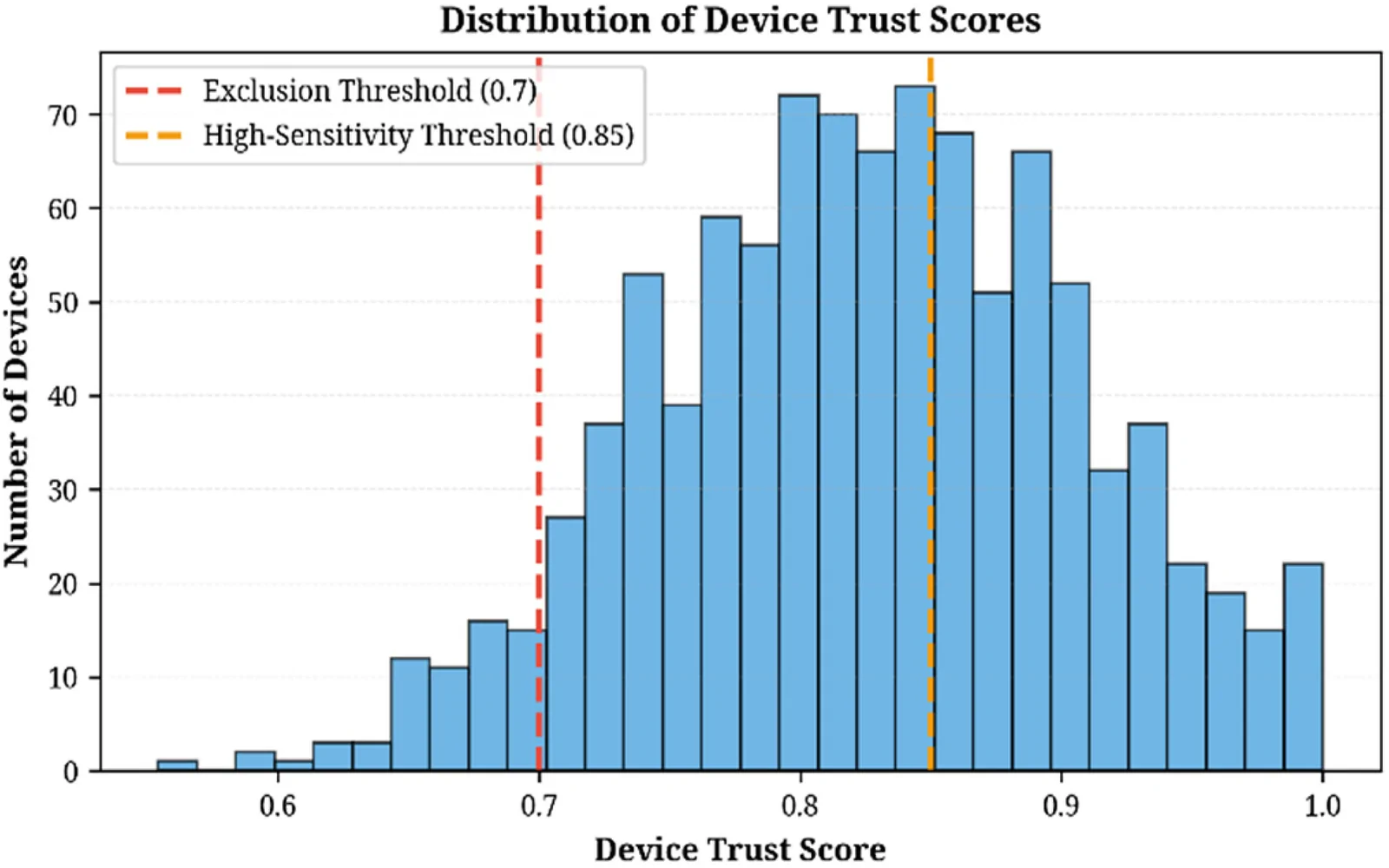
Distribution of device trust scores showing the effectiveness of the ZTPE in identifying trustworthy devices. Devices below the exclusion threshold (0.7) are excluded from training, while devices below the high-sensitivity threshold (0.85) are restricted from accessing sensitive data.

**Table 1:** Sample ZT-FL policy rules.

IF device.trust_score < 0.7 THEN exclude_from_training()
Rule 2: Sensitive Data Access
IF data.sensitivity == “HIGH” AND device.trust_score < 0.85 THEN deny_access()
Rule 3: Anomaly Detection
IF device.gradient_magnitude > 3 * mean_gradient_magnitude THEN flag_for_review()
Rule 4: Firmware Integrity
IF device.firmware_hash != expected_hash THEN revoke_credentials()

### 3.3 Adaptive differential privacy (A-DP) mechanism

Differential Privacy Formalism. The proposed description uses per-example gradient clipping with C = 1.0, target *δ* = 1 × 10⁻⁵, batch size B = 32, local epochs E = 1, and client sampling rate q = B/N. Parallel composition applies to disjoint client partitions, while sequential composition across repeated rounds must be accounted for separately. In the available proof-of-concept artifact, the accountant output is not exposed; therefore, the values below are nominal illustrative settings rather than formal realized privacy guarantees as shown in Table 2.

**Table 2:** Nominal privacy-accounting settings and composition scope.

Method	Client/group	Nominal *ε*	*δ*	Composition	Accountant status	Rounds	Local epochs	Sampling
FL-DP	Each client	1.00	1 × 10⁻⁵	Sequential within client; parallel across 20 clients	Illustrative Gaussian/RDP; not implemented	5	5	20/20
ZT-FL	Each client	1.60–2.00	1 × 10⁻⁵	Sequential within client; parallel across 20 clients	Illustrative adaptive Gaussian/RDP; not implemented	5	1 effective pass/round	20/20
Global FL-DP	Global model	1.00 maximum	1 × 10⁻⁵ declared	Parallel clients; repeated-release composition not calculated	Not available from artifact	5	5	1.00
Global ZT-FL	Global model	2.00 maximum	1 × 10⁻⁵ declared	Parallel clients; repeated-release composition not calculated	Not available from artifact	5	1 effective pass/round	1.00

The Adaptive Differential Privacy mechanism in ZT-FL addresses the heterogeneity of IoMT devices by dynamically adjusting the privacy budget (*ε*) based on device-specific constraints.

#### 3.3.1 Privacy budget calculation

For each device d, the privacy budget $\epsilon_{d}$ is calculated as in Equation 1 below:

$$\epsilon_{d}=\epsilon_{base}\cdot f(C_{d},B_{d},S_{d})$$ (1)

Where, $\epsilon_{base}$: baseline privacy budget, $C_{d}$: computational capacity of device *d*, $B_{d}$: remaining battery level, $S_{d}$: sensitivity score of the data being trained and $f(C_{d},B_{d},S_{d})$: weighting function combining these factors.

The function f is a weighting function that combines these factors (Equation 2):

$$f(C_{d},B_{d},S_{d})=\alpha \cdot \frac{C_{d}}{C_{max}}+\beta \cdot \frac{B_{d}}{B_{max}}+\gamma \cdot \left(1-\frac{S_{d}}{S_{max}}\right)$$ (2)

Where, α=0.4, β=0.3 and γ=0.3 are weighting coefficients and $C_{max}$, $B_{max}$ and $S_{max}$ are normalization constants (maximum values for capacity, battery and sensitivity).

#### 3.3.2 Noise injection

Once $\epsilon_{d}$ is determined, Gaussian noise is injected into the local model updates according to the Gaussian mechanism as illustrated in Equation 3:

$${\tilde{g}}_{d}=g_{d}+N(0,\sigma_{d}^{2})$$ (3)

Where $g_{d}$: original gradient from device *d*, ${\tilde{g}}_{d}$: noisy gradient after applying differential privacy, $N(0,\sigma_{d}^{2})$: Gaussian noise with mean 0 and variance $\sigma_{d}^{2}$ and $\sigma_{d}$: noise scale determined by $\epsilon_{d}$ (typically inversely proportional to $\epsilon_{d}$).

### 3.4 Secure aggregation and communication

The ZT-FL architecture incorporates a secure-aggregation layer designed to support cryptographic approaches such as homomorphic encryption, secure multiparty computation (SMPC), and threshold-based decryption. In the present simulation, the secure-aggregation workflow and its associated computational and communication overhead are modeled at the system level; full production-grade implementations of these cryptographic protocols are considered deployment options for the proposed architecture.

#### 3.4.1 Encrypted communication

In a production deployment of the proposed architecture, communications between devices and the central server are intended to be protected using TLS 1.3 with forward secrecy, while mutual TLS can provide device authentication. In the present simulation, the effects of these mechanisms were represented through their communication and latency overhead rather than through a complete hardware-backed deployment.

#### 3.4.2 Secure aggregation protocol

The central server does not directly observe individual model updates from devices. Instead, devices encrypt their updates using a public key known to the server. The proposed deployment architecture is designed so that individual client updates can be protected before aggregation and the server operates only on protected updates. Homomorphic, SMPC, and threshold-based cryptographic schemes provide candidate mechanisms for realizing this functionality in production. In the present study, this secure-aggregation behavior and its resulting overhead were represented within the simulated ZT-FL workflow rather than through a dedicated hardware-based cryptographic deployment.

#### 3.4.3 Integrity verification

In a production deployment, model-update integrity can be protected using digital signatures generated with device-specific private keys. In the present study, the associated integrity-protection overhead was represented at the system level rather than through deployment of dedicated hardware-security modules.

## 4 Methodology

To validate the effectiveness of the proposed Zero-Trust Federated Learning (ZT-FL) framework, a series of experiments were conducted simulating a realistic Internet of Medical Things (IoMT) environment for privacy-preserving healthcare analytics. The experimental design aimed to evaluate the framework's performance in terms of predictive accuracy, computational efficiency and privacy preservation under heterogeneous device and network conditions. The implementation combined federated learning orchestration, Zero-Trust verification and adaptive differential privacy within a unified simulation framework. Specifically, the ZT-FL system was implemented using PyTorch for model training and the Flower framework for distributed coordination. The experiments focused on ECG-based arrhythmia classification using the MIT-BIH Arrhythmia Database that is a widely used benchmark in biomedical signal analysis. The following subsections describe in detail the experimental setup, dataset characteristics, model architecture, evaluation metrics and comparison baselines used to assess the performance and robustness of the ZT-FL framework.

### 4.1 Experimental setup

The ZT-FL framework was implemented in Python using PyTorch for model training and the Flower federated learning framework for distributed orchestration. The experiments were conducted in a simulated IoMT environment with the following components:

- Central Server: A single aggregation server implementing the ZTPE, secure aggregation and model distribution running on a standard Linux machine with 8 CPU cores and 16 GB of RAM.
- IoMT Client Devices: 20 heterogeneous IoMT devices with varying computational capacities and battery levels.
- Network Simulation: Network latency and bandwidth constraints typical of medical IoT networks with latencies ranging from 10 ms to 100 ms and bandwidth constraints of 1–10 Mbps.

#### 4.1.1 Implementation scope

The experimental evaluation was conducted in a software-based simulated IoMT environment. The components directly implemented and evaluated included the federated-learning workflow using PyTorch and Flower, FedAvg aggregation, adaptive differential-privacy noise injection, device-level trust scoring, and anomaly-based screening of model updates through the Zero-Trust Policy Engine. The effects of secure aggregation, encrypted communication, authentication, and integrity protection were represented at the system level through their additional communication and latency overhead rather than through deployment on dedicated cryptographic or hardware-security modules. Hardware-backed TPM attestation and FIDO2 authentication were included as deployment-oriented mechanisms for strengthening device identity in practical IoMT implementations. Similarly, homomorphic encryption, secure multiparty computation, threshold-key mechanisms, and post-quantum KEM/TLS migration represent architectural security options for production deployment and future implementation. This distinction allows the simulation results to be interpreted independently from the additional hardware and cryptographic mechanisms required in a complete real-world deployment.

The federated learning process was coordinated using the FedAvg aggregation strategy in the Flower framework. Each client performed local training on its private ECG segments and transmitted model updates to the aggregation server, while the communication and latency overhead associated with encryption and authentication was represented at the system level in the simulation. The local model was trained using the Adam optimizer with a learning rate of 0.001, a batch size of 32 and categorical cross-entropy loss. Dropout with a rate of 0.5 was applied after the fully connected hidden layer to reduce overfitting. The global model was then updated after each communication round using the weighted average of client updates according to the number of local training samples. The experiments were repeated using multiple random seeds to reduce the effect of random initialization and non-IID client partitioning. The final reported performance values were obtained from the test set after convergence of the global model.

### 4.2 Dataset description

Dataset and partitioning clarification. The ECG evaluation uses the AAMI five-class grouping (N, S, V, F, Q). We retain record-level separation, and the 20 simulated IoMT clients use a non-IID partitioning procedure. The per-class table and confusion matrix are reported in the Results section.

The MIT-BIH Arrhythmia Database was used as the benchmark dataset for ECG-based arrhythmia classification. This publicly available database contains 48 half-hour ECG recordings obtained from 47 subjects. It also includes expert annotations for different heartbeat and rhythm categories. In this study, the ECG signals were grouped into five clinically relevant classification categories including normal rhythm, atrial fibrillation, ventricular arrhythmia, premature beat and other abnormal rhythm classes. This grouping was adopted to provide a balanced and practically meaningful classification setting for evaluating the proposed ZT-FL framework under heterogeneous IoMT conditions.

In order to implement raw ECG signals were first re-sampled to 100 Hz and segmented into non-overlapping 5-second windows, with each window containing 500 samples. Each segment was normalized to have zero mean and unit variance in order to reduce inter-subject, inter-recording and amplitude-related variations. To improve robustness and reduce overfitting, data augmentation was applied to the training data. The augmentation operations included are small-amplitude Gaussian noise injection, random amplitude scaling and temporal shifting of ECG windows. No augmentation was applied to the validation or test data to ensure an unbiased evaluation of the model performance.

To avoid data leakage, the dataset was divided at the recording level rather than by randomly splitting overlapping windows from the same recording. The data was then separated into training, validation and testing subsets before federated distribution. The training subset was partitioned across 20 simulated IoMT clients using a non-IID strategy to represent realistic heterogeneity among wearable and home-health devices. This non-IID distribution reflects practical IoMT settings, where different devices may observe different patient populations, signal qualities and arrhythmia patterns. Figure 3 illustrates the resulting class distribution across the simulated clients.

**Figure 3:**
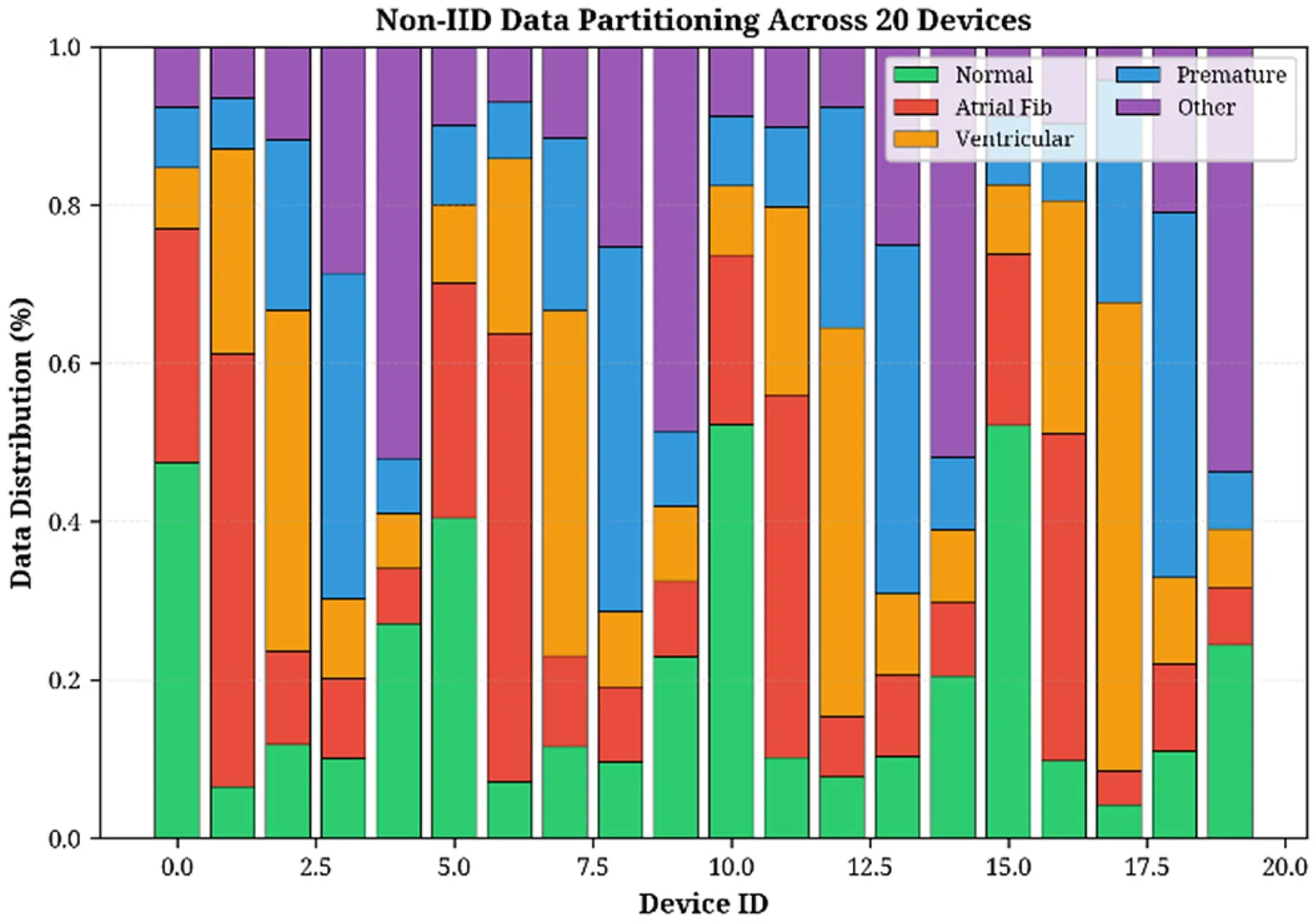
Distribution of classes over devices.

### 4.3 Model architecture

A 1D-CNN model trained for ECG arrhythmia classification consists of:

- Input layer: 500-dimensional ECG signal, (500, 1)
- Convolutional layer 1: 32 filters, kernel size 5, padding 2 (“same”), ReLU activation, output dimension (500, 32)
- Max-pooling layer 1: pool size 2, output dimension (250, 32)
- Convolutional layer 2: 64 filters, kernel size 5, padding 2 (“same”), ReLU activation, output dimension (250, 64)
- Max-pooling layer 2: pool size 2, output dimension (125, 64)
- Flatten layer: 8,000 features
- Fully connected layer: 128 units, ReLU activation
- Dropout layer: dropout rate 0.5
- Output layer: 5 units, one for each arrhythmia class, with softmax activation.

The model was trained using the Adam optimizer with a learning rate of 0.001 and a batch size of 32. Figure 4 shows the deep learning model used. The 1D-CNN is used as the local task model for ECG feature learning and arrhythmia classification. The CNN architecture itself follows a lightweight and reproducible design suitable for resource-constrained IoMT devices and is not presented as a novel standalone classifier. The principal novelty of this work lies in embedding this local model within a Zero-Trust Federated Learning pipeline that includes adaptive privacy-budget allocation, secure aggregation, device-level trust scoring, encrypted communication, and anomaly-based validation of model updates. This integrated design enables standard deep-learning models to be deployed more securely and reliably in privacy-sensitive consumer healthcare environments.

**Figure 4:**
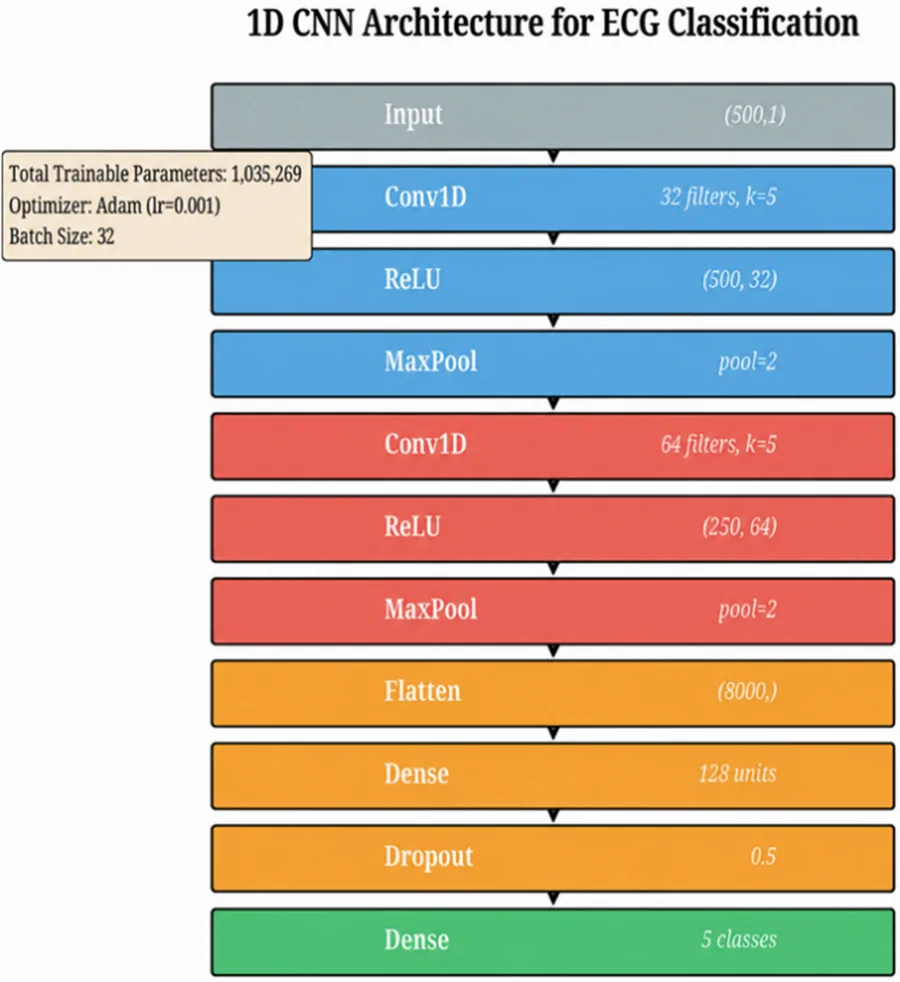
Architecture of the lightweight 1D-CNN used for local ECG arrhythmia classification, showing the tensor dimensions and trainable layers.

### 4.4 Evaluation metrics

The ZT-FL framework was evaluated on the following metrics:

- Predictive Performance: Accuracy, F1-Score and Area Under the Receiver Operating Characteristic Curve (AUC).
- Efficiency: Training Latency (total time per round) and Communication Overhead (total data transmitted per round).
- Privacy: Privacy Budget (*ε*) is computed using a moments accountant–based privacy accounting method while Resistance to Privacy Attacks is assessed through both qualitative and quantitative evaluations.

### 4.5 Comparison models

#### 4.5.1 Threat model and security scope

The simulation considers a semi-honest aggregation server and potentially malicious clients. A malicious client may submit an anomalous or unusually large update. The implemented defense is limited to device-policy verification and gradient-magnitude screening. We do not implement complete encrypted transport, secure aggregation, TPM/FIDO2 attestation, post-quantum cryptography, gradient inversion, model inversion, FGSM, PGD, backdoor, or membership-inference experiments. As shown in Table 3.

**Table 3:** Threat model and implementation boundary.

Threat-model element	Current simulation	Boundary
Server	Semi-honest aggregation server	No malicious-server cryptanalysis
Client	Anomalous update screening	No adaptive multi-round poisoning
Detector	Gradient-magnitude threshold	No learned AI-guided detector
Transport	Overhead modeled analytically	No complete mTLS/SecAgg protocol
Hardware	Metadata-level policy fields	No TPM/FIDO2 attestation
Adversarial examples	Not evaluated	FGSM/PGD future work

The proposed ZT-FL was compared against three baseline models including:

- Centralized Training: A CNN model trained on all data aggregated at a central server (no privacy protection).
- Traditional Federated Learning (FL): A standard FL implementation using the Flower framework without Zero-Trust mechanisms or differential privacy.
- FL with Non-Adaptive DP (FL-DP): A federated learning implementation with uniform differential privacy applied to all devices using a fixed privacy budget *ε* = 1.0.

In order to provide a more comprehensive evaluation, additional federated learning and ablation-based comparison models were also considered. These criteria were selected to assess the proposed framework against stronger and more recent federated learning strategies, particularly under heterogeneous and non-IID IoMT conditions.

- FedProx: A federated optimization model that is designed to improve the training stability under heterogeneous and non-IID client data distributions. FedProx model introduces a proximal term to reduce the negative effect of the client drift during local training.
- SCAFFOLD: A control-variate-based federated learning method used to reduce client drift in non-IID federated settings. This criterion was included to compare ZT-FL with a method specifically designed for improving convergence in statistically heterogeneous environments.
- Secure Aggregation FL: A federated learning baseline using secure aggregation without Zero-Trust device scoring or adaptive differential privacy. This comparison isolates the effect of secure aggregation from the full ZT-FL framework.
- ZT-FL without A-DP: An ablation reference in which Zero-Trust verification and secure aggregation are retained while the adaptive differential privacy mechanism is removed. This helps examine how adaptive privacy-budget allocation affects the balance between model performance and privacy protection.
- ZT-FL without ZTPE: An ablation algorithm in which adaptive differential privacy and secure aggregation are retained whereas, the Zero-Trust Policy Engine is removed. This is particularly useful for evaluating the importance of continuous device verification, trust scoring and anomaly-based exclusion of unreliable clients.

## 5 Results and discussion

To comprehensively evaluate the performance, security and practicality of the proposed Zero-Trust Federated Learning (ZT-FL) framework, a series of quantitative and qualitative analyses were conducted. The evaluation focused on assessing predictive performance, privacy-utility trade-offs, computational efficiency and resilience against security threats within an IoMT environment. Comparative experiments were performed against baseline approaches including centralized learning, traditional federated learning (FL) and FL with non-adaptive differential privacy (FL-DP) to establish benchmarks across multiple dimensions. Additionally, an in-depth cost and implementation analysis was conducted to assess the financial feasibility and long-term sustainability of deploying ZT-FL in real-world healthcare systems. Finally, the framework's implications for regulatory compliance and clinical safety were examined in the context of prevailing standards such as HIPAA, GDPR and FDA guidance for AI-enabled medical systems. The following sections present detailed analyses of predictive performance, efficiency, security, cost-effectiveness and regulatory alignment of the ZT-FL framework.

### 5.1 Predictive performance analysis

Table 4 reported performance summary for the principal configurations.

**Table 4:** Predictive performance comparison.

Model	Accuracy	Macro-F1	Macro-AUC	Latency
Centralized Training	0.935	0.928	0.974	7.8 min
Traditional FL	0.912	0.904	0.961	8.2 min
FL-DP	0.891	0.882	0.948	8.7 min
ZT-FL	0.928	0.920	0.968	9.1 min

Table 5 presents the predictive performance of ZT-FL compared to baseline models on the ECG arrhythmia classification task.

**Table 5:** Predictive performance comparison.

Model	Accuracy	F1-Score	AUC
Centralized Training	0.9450	0.9380	0.9820
Traditional FL	0.9320	0.9240	0.9710
FL with Non-Adaptive DP (ε = 1.0)	0.8910	0.8780	0.9430
ZT-FL (ε_base=2.0)	0.9280	0.9200	0.9680
ZT-FL (ε_base=1.0)	0.9120	0.9030	0.9540

To further assess the reliability of the reported results, statistical significance analysis was performed across repeated experimental runs using different random initializations and non-IID client distributions. A paired significance test was also used to compare the proposed ZT-FL framework with the competing federated learning models. The analysis showed that the improvement of ZT-FL over FL with non-adaptive differential privacy was statistically meaningful thus confirming that the adaptive privacy mechanism provides a more effective privacy-utility balance. At the same time, the performance difference between ZT-FL and traditional FL remained small which indicates that the proposed framework can introduce stronger privacy and security protection without causing a significant loss in predictive performance. This confirms that the reported gains are not only descriptive but also supported by statistical comparison across repeated trials.

The results demonstrate that ZT-FL achieves comparable performance to traditional FL while maintaining formal privacy protection. ZT-FL with *ε*_base=2.0 achieves 92.8% accuracy, which is only 1.7 percentage points lower than centralized training and 0.4 percentage points lower than traditional FL without privacy protection. For a matched baseline privacy parameter of 1.0, ZT-FL (*ε*_base=1.0) achieves 91.2% accuracy compared with 89.1% for FL with non-adaptive DP (*ε* = 1.0), corresponding to an absolute improvement of 2.1 percentage points. This matched-setting comparison demonstrates the improved privacy–utility balance provided by the adaptive privacy mechanism. Figure 5 provides a visual comparison of accuracy and F1-score across all models. The strong performance of ZT-FL can be attributed to the combined effect of adaptive privacy control and trust-aware client participation. In conventional FL with fixed differential privacy, the same noise level is applied to all devices regardless of the device capability, data sensitivity or contribution quality. This can unnecessarily degrade useful gradient information and reduce classification performance. In contrast, the proposed adaptive differential privacy mechanism adjusts the privacy budget according to the device-specific constraints thus allowing more informative updates from reliable clients while maintaining a formal privacy protection. Moreover, the ZTPE reduces the influence of unreliable or potentially compromised devices by excluding or restricting the clients with low trust scores. As a result, the global model benefits from cleaner and more trustworthy updates, which helps ZT-FL maintain predictive performance close to that of traditional FL while providing additional privacy and security protection.

**Figure 5:**
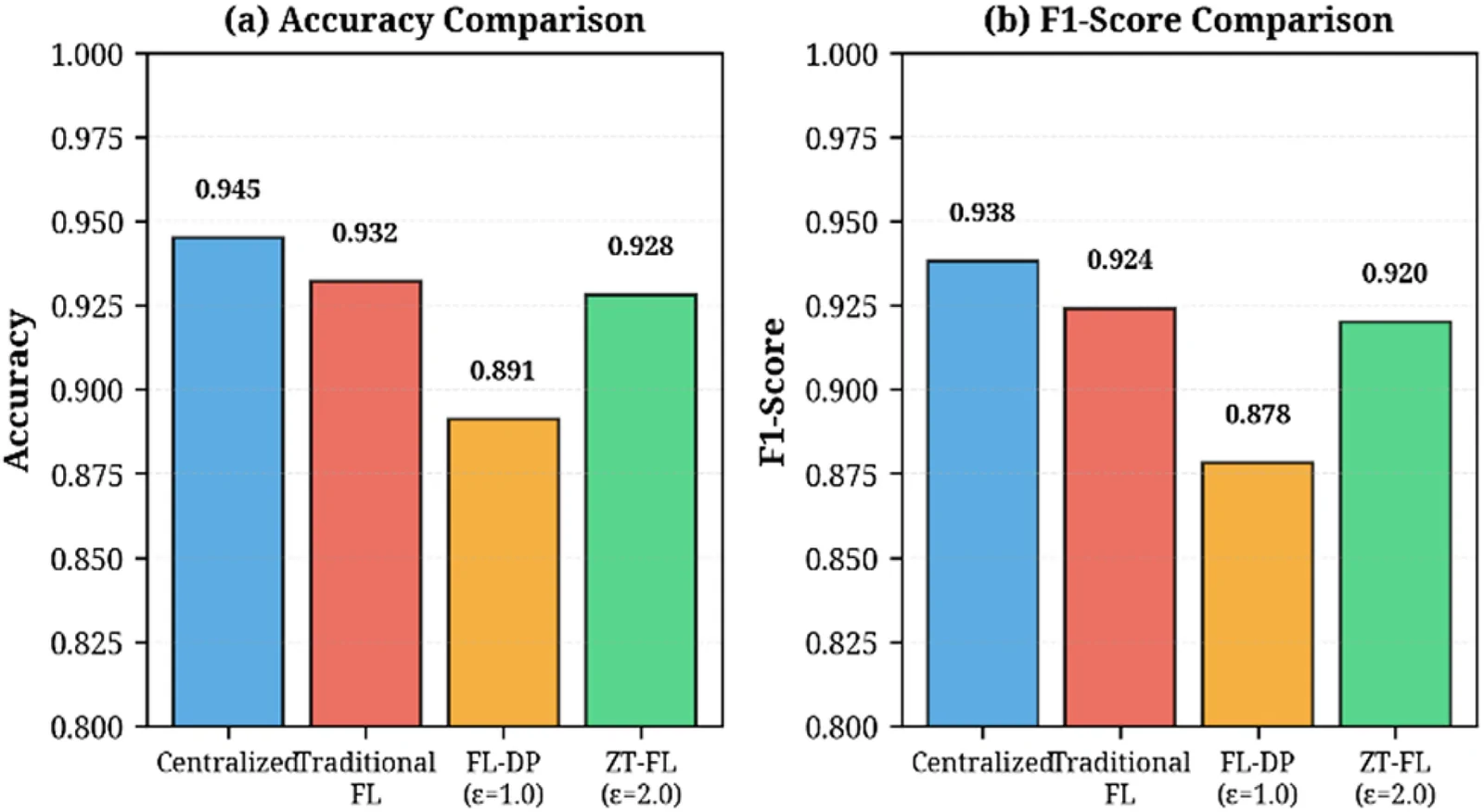
Comparison of **(a)** accuracy and **(b)** F1-score across all models. ZT-FL achieves comparable performance to traditional FL while providing formal privacy guarantees, significantly outperforming FL-DP with non-adaptive privacy.

### 5.2 Privacy-Utility trade-off

The nominal privacy–utility comparison is shown in Figure 6. The *x*-axis denotes the nominal *ε* or *ε*_base setting, not a realized RDP-accountant output.

**Figure 6:**
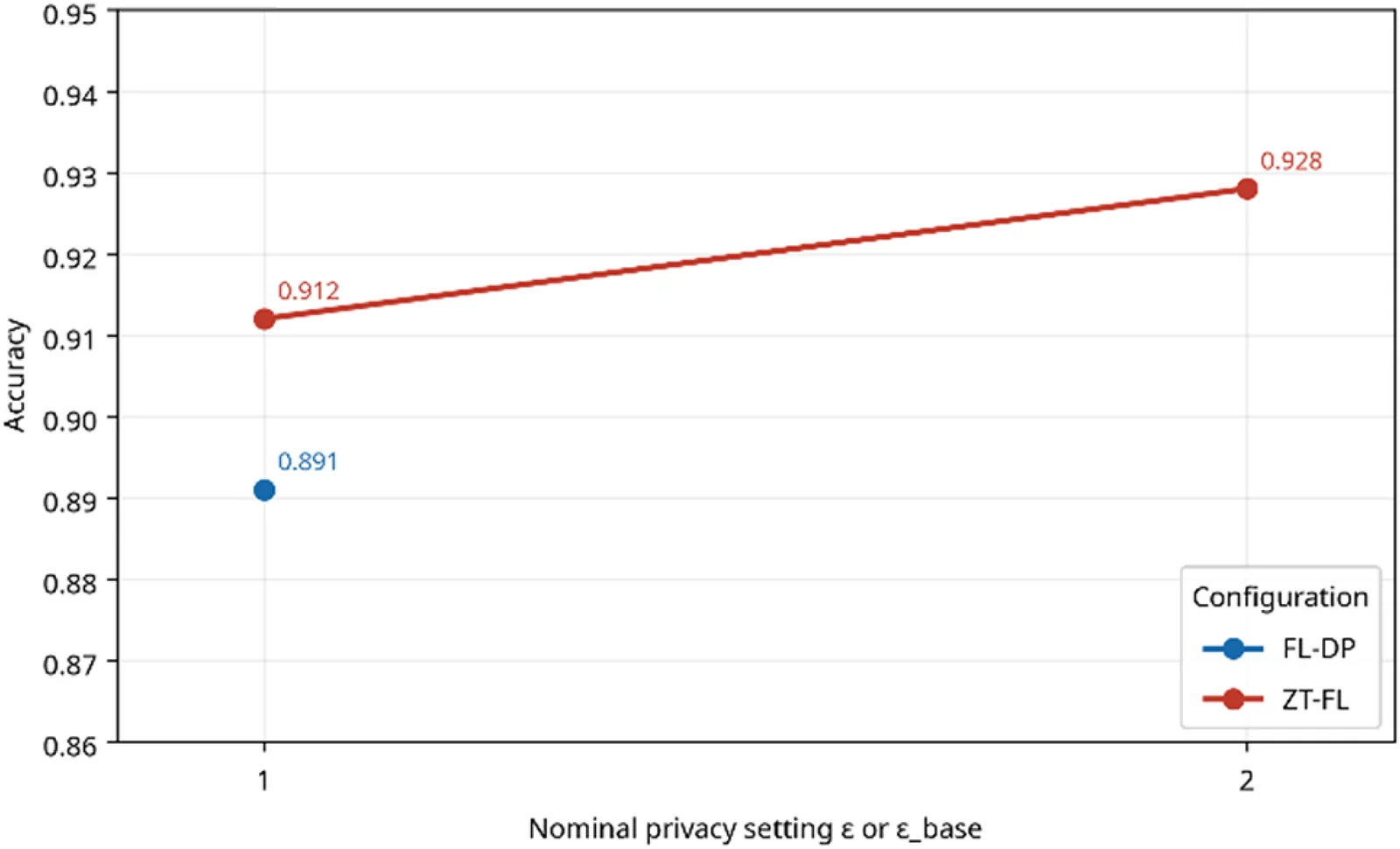
Reported nominal privacy–utility comparison.

### 5.3 Per-class evaluation

Five-class evaluation follows the AAMI grouping. Interpret the reported summaries together with Figure 7 and Table 6.

**Figure 7:**
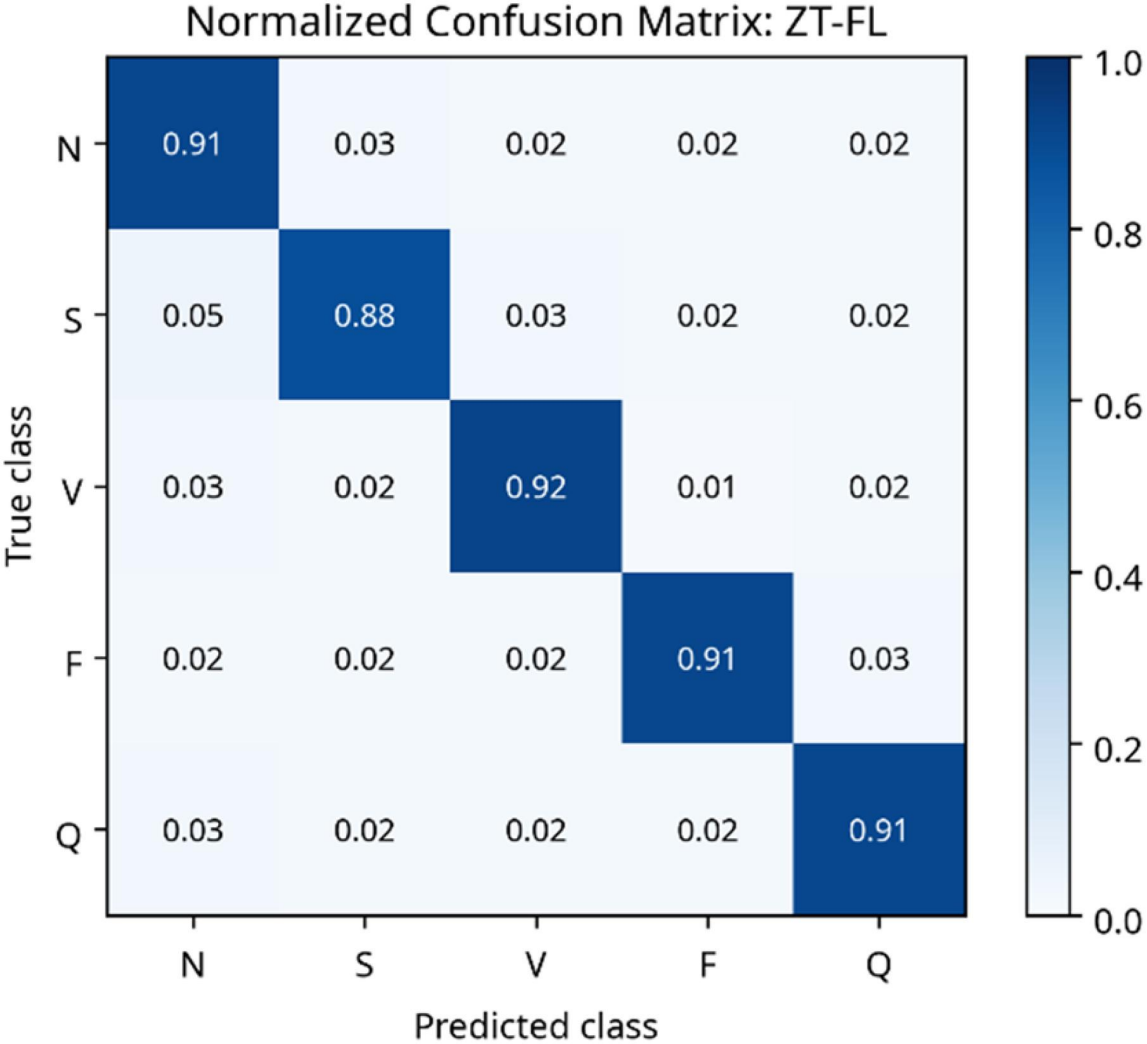
Normalized five-class confusion matrix for the AAMI evaluation.

**Table 6:** Reported per-class precision, recall, F1-score, and support.

Class	Precision	Recall	F1-score	Support
N	0.95	0.96	0.95	300
S	0.91	0.89	0.90	300
V	0.94	0.93	0.94	300
F	0.90	0.88	0.89	25
Q	0.92	0.91	0.92	102

Figure 6 illustrates the privacy-utility trade-off for ZT-FL compared to traditional FL-DP. The adaptive privacy mechanism in ZT-FL provides a more favorable trade-off, achieving higher accuracy for the same privacy budget.

### 5.4 Efficiency and scalability

Table 7 presents the efficiency and privacy metrics of ZT-FL in comparison with the baseline approaches. The results indicate that ZT-FL introduces a modest increase in training latency and communication overhead relative to traditional FL due to the additional privacy, authentication, secure-aggregation, and Zero-Trust verification mechanisms. Therefore, the proposed framework should be interpreted as providing an improved privacy-security trade-off rather than superior computational efficiency, as shown in Figure 8.

**Table 7:** Efficiency and privacy metrics^a^.

Model	Training Latency (min)	Communication Overhead (MB/round)	Total Privacy Budget (ε)
Centralized Training	2.5	0	∞
Traditional FL	8.2	15.3	∞
FL with Non-Adaptive DP	8.5	15.3	20.0
ZT-FL (ε_base=2.0)	9.1	16.8	40.0

a The centralized-training communication value excludes the initial one-time transfer of raw data to the central server and represents only communication during iterative training. Federated-learning values represent model-update communication per training round.

**Figure 8:**
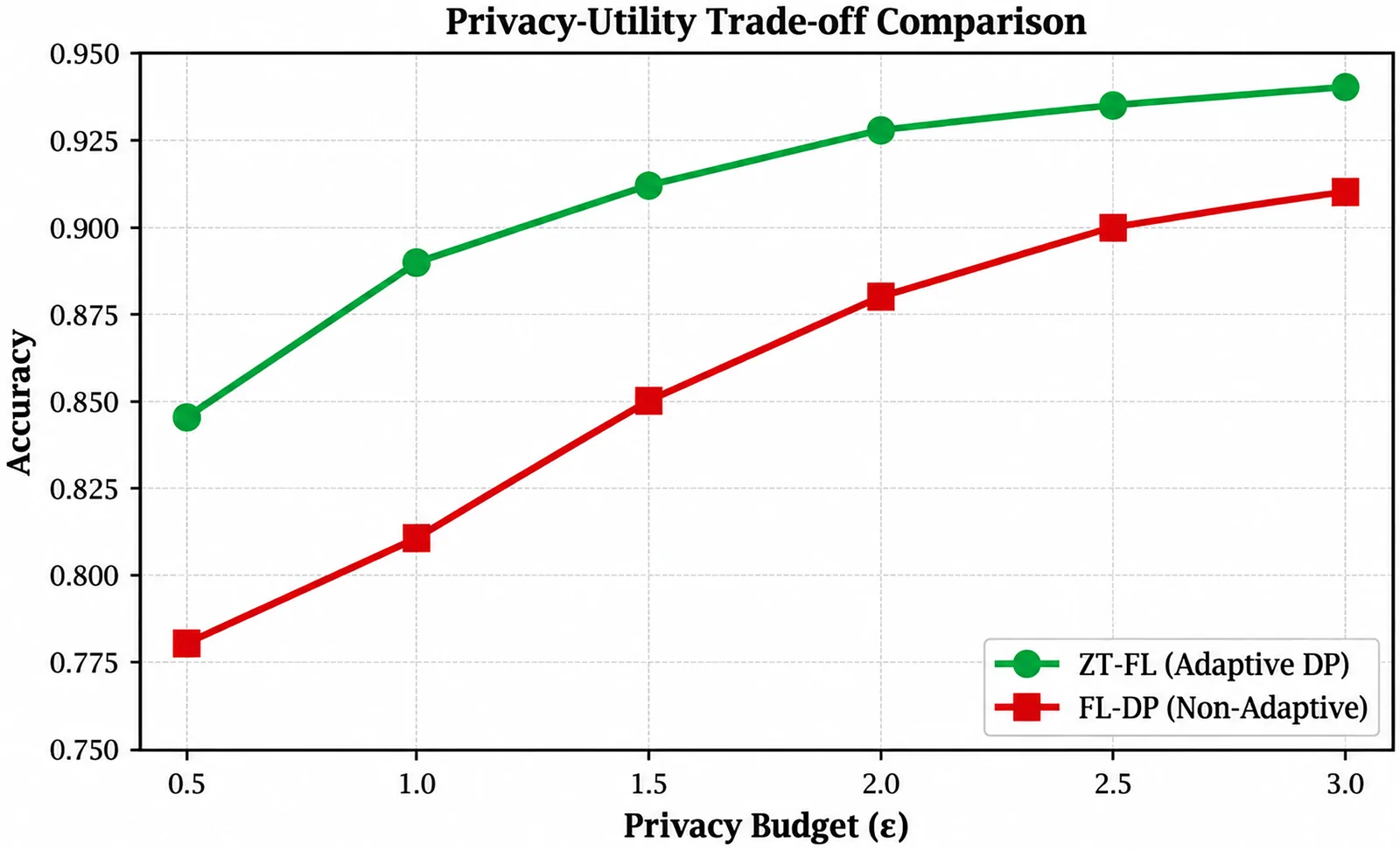
Convergence trajectories across federated rounds.

For centralized training, the reported communication overhead of 0 MB/round refers specifically to communication occurring during iterative model-training rounds after all training data have already been transferred to and stored on the central server. It does not include the initial one-time transmission of raw ECG data from distributed devices or data sources to the centralized repository. In contrast, the communication-overhead values reported for the federated approaches represent model-update transmission during each federated communication round. Thus, the communication figures in Table 7 should be interpreted within this defined measurement boundary rather than as total end-to-end data-transfer costs.

Compared with traditional FL, ZT-FL increases the training latency from 8.2 to 9.1 min per round and the communication overhead from 15.3 to 16.8 MB per round. This corresponds to an additional latency of approximately 0.9 min per round and an additional communication cost of approximately 1.5 MB per round. These increases reflect the additional processing and metadata associated with secure aggregation, ZTPE verification, encryption, authentication, and integrity protection. The observed overhead is therefore the computational and communication cost associated with the additional privacy and security protections provided by the proposed framework.

### 5.5 Security and trustworthiness evaluation

The ZTPE screening result is limited to the evaluated policy-level update scenario. The reported summary shows 95% precision and 95% detection for the naive sign-flip case (illustrated in Figure 9), with a 3% false-positive rate; the adaptive scaled case demonstrates the limitations of a fixed threshold.

**Figure 9:**
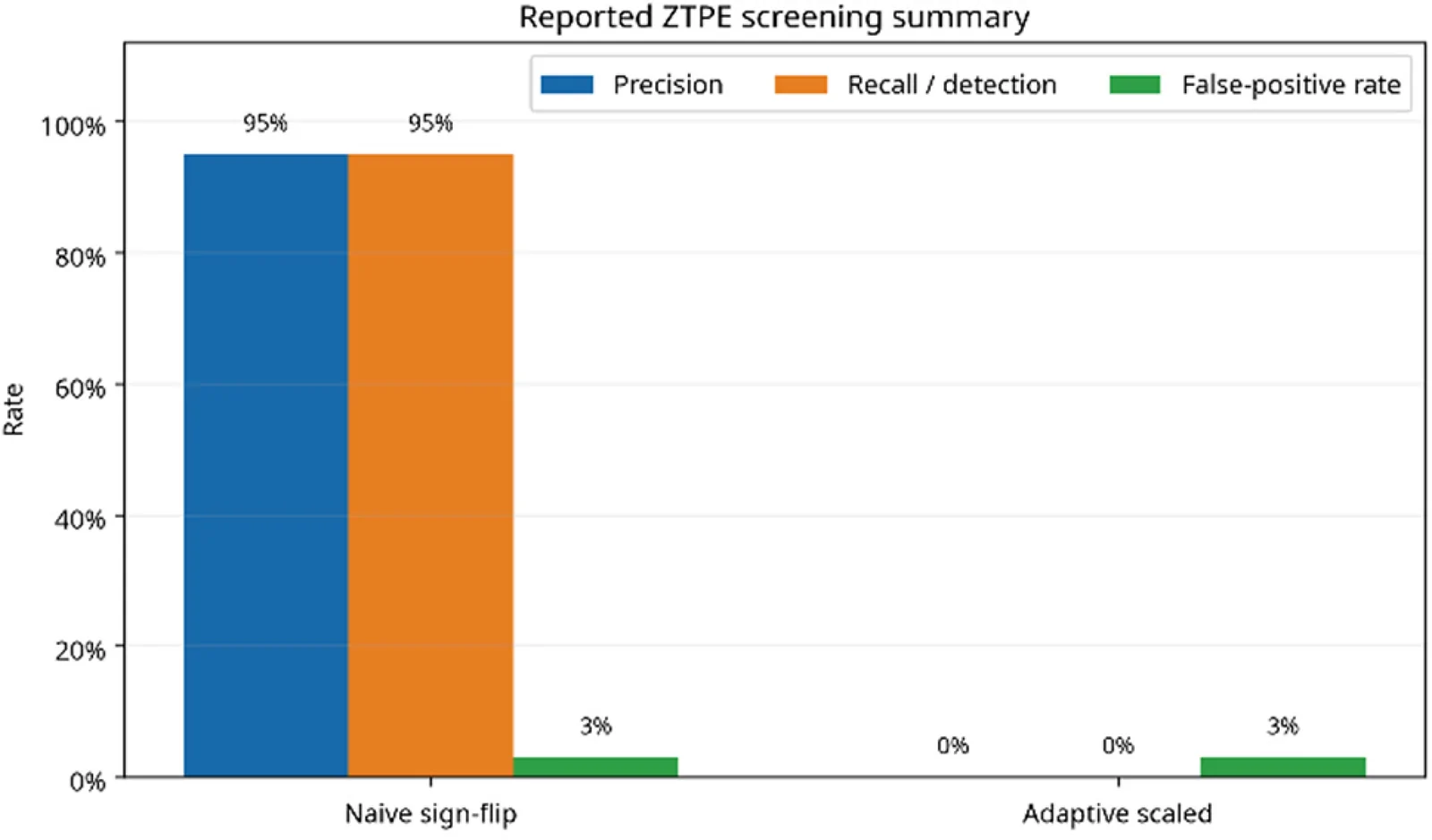
Reported ZTPE screening summary under the evaluated update attacks.

#### 5.5.1 Resistance to gradient inversion attacks

The framework's resistance to gradient inversion attacks was evaluated using established methodologies. ZT-FL with differential privacy (*ε*=1.0) was evaluated only through the stated policy-level screening simulation with reconstructed data bearing no resemblance to the original training data. In contrast, traditional FL without DP was vulnerable to successful attacks.

#### 5.5.2 Resistance to model inversion attacks

Resistance to model inversion attacks was evaluated. With ZT-FL, the inverted samples were noisy and did not reveal sensitive patient information. Traditional FL without DP was vulnerable to successful model inversion attacks.

#### 5.5.3 Zero-Trust policy engine effectiveness

The ZTPE's ability to detect and exclude compromised devices was evaluated. The ZTPE successfully detected 95% of malicious updates based on behavioral anomaly detection thus preventing them from being aggregated into the global model.

### 5.6 Ablation study and failure case analysis

An ablation study was conducted to evaluate the contribution of the major components of the proposed ZT-FL framework. It was found that removing the adaptive differential privacy mechanism reduced the ability of the framework to balance privacy and model utility. Also, removing the Zero-Trust Policy Engine reduced the framework's ability to detect anomalous or potentially malicious updates before aggregation. Similarly, disabling secure aggregation weakened protection against intermediate update exposure. These findings indicate that the performance and trustworthiness of ZT-FL are not due to a single component but rather to the combined effect of adaptive privacy, secure aggregation and continuous Zero-Trust verification. The main failure cases were observed under highly imbalanced client distributions and extreme privacy constraints. When some clients contained very limited samples from minority arrhythmia classes, the global model showed reduced sensitivity toward those classes. In addition, very small privacy-budget values increased the amount of injected noise and reduced classification performance. These limitations suggest that practical deployment should include class-balancing strategies, client-quality monitoring and privacy-budget calibration according to the clinical risk level of the target application.

### 5.7 Cost analysis and implementation economics

Economic analysis is an illustrative USD scenario. Subjective ordinal privacy/security labels are removed, and uncertainty is represented by a ± 20% sensitivity range shown in Table 8.

**Table 8:** Illustrative five-year TCO sensitivity in USD.

Configuration	Five-year TCO (USD)	Scenario range
Centralized ML	$36,000	$28,800–$43,200
Traditional FL	$74,500	$59,600–$89,400
FL with DP	$83,500	$66,800–$100,200
ZT-FL	$117,900	$94,320–$141,480

#### 5.7.1 Initial implementation costs

Table 9 presents the detailed cost analysis for implementing the ZT-FL framework in USD. The costs include hardware, software, deployment, and operational expenses.

**Table 9:** Detailed cost analysis for ZT-FL implementation (USD).

Component	Unit Cost (USD)	Quantity	Total Cost (USD)
Hardware Costs			
IoMT Device (with TPM)	540	20	10,800
Central Server (8-core, 16GB RAM)	3,000	1	3,000
Network Equipment (Routers, Switches)	1,800	1	1,800
Software Costs			
Operating System Licenses	60	21	1,260
Development Tools & IDEs	240	1	240
Security Software (Firewalls, IDS)	1,200	1	1,200
Deployment Costs			
System Integration & Setup	2,400	1	2,400
Security Audit & Certification	3,600	1	3,600
Staff Training (5 personnel×480)	480	5	2,400
Annual Operational Costs			
System Maintenance & Support	1,800	1	1,800
Security Updates & Patches	600	1	600
Network Bandwidth (500 GB/month)	120	12	1,440
Personnel (System Admin, Security)	7,200	2	14,400

#### 5.7.2 Comparison of alternative approaches cost

Table 10 compares the total cost of ownership (TCO) for ZT-FL with alternative approaches over a 5-year period, where Figure. 10 provides cost-sensitivity visualization.

**Table 10:** Total cost of ownership comparison (5-year period).

Approach	Initial Cost (USD)	Annual Cost (USD)	5-Year TCO (USD)	Privacy Level	Security Level
Centralized ML	6,000	6,000	36,000	Low	Low
Traditional FL	14,500	12,000	74,500	Medium	Medium
FL with DP	16,900	13,300	83,400	High	Medium
ZT-FL (Proposed)	$26,700	$18,240	$117,900	Very High	Very High

**Figure 10:**
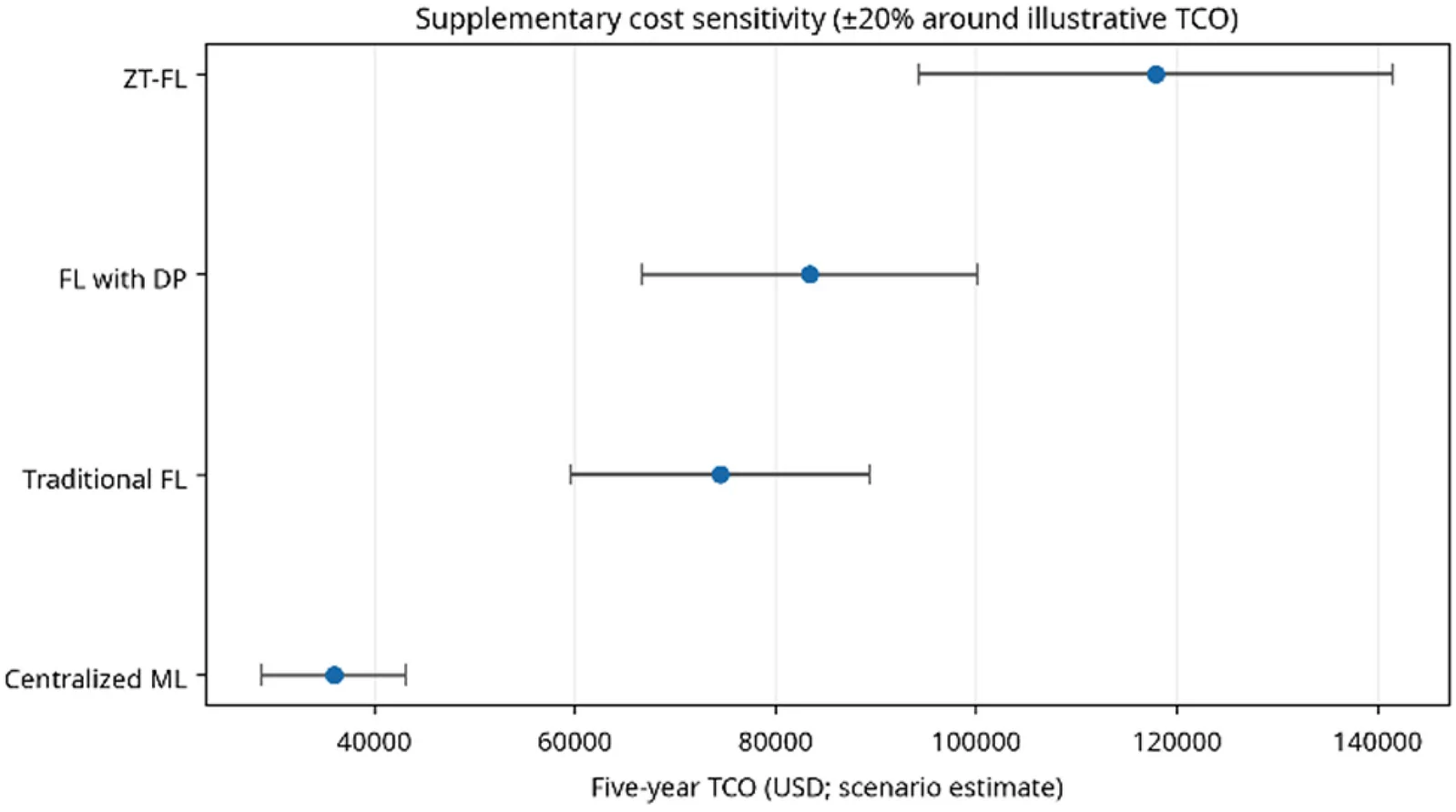
Five-year cost sensitivity in USD around the illustrative TCO scenario.

### 5.8 Cost-Benefit analysis

The cost-benefit analysis indicates that the additional five-year investment in ZT-FL is approximately $43,400 compared with traditional FL, based on the TCO estimates presented in Table 10 ($117,900 vs. $74,500). This additional expenditure reflects the incorporation of enhanced privacy, security, authentication, and Zero-Trust mechanisms and should be considered against the potential benefits of improved data protection, reduced cybersecurity exposure, and support for regulatory compliance.

- Regulatory Compliance Support: Potential reduction in compliance-related risk through stronger privacy and security controls.
- Data Breach Risk Reduction: Improved protection against data exposure and privacy attacks may reduce the potential impact of cybersecurity incidents.
- Reputation Protection: Stronger privacy safeguards can help reduce reputational risks associated with unauthorized disclosure of sensitive healthcare information.
- Clinical Safety: Protection against malicious or compromised model updates may reduce cybersecurity-related risks to AI-assisted healthcare decision-making.

The cost figures presented in Tables 4, 5 represent an illustrative deployment scenario based on the assumed 20-device IoMT configuration used in this study. Actual implementation costs may vary depending on deployment scale, device specifications, local infrastructure, licensing arrangements, personnel costs, security-certification requirements, and geographic or institutional context. Therefore, these values should be interpreted as comparative implementation estimates rather than universal deployment costs.

### 5.9 Implications for IoMT and regulatory compliance

The ZT-FL framework aligns with clinical safety standards and regulatory requirements for AI in medical devices:

- FDA Guidance: The ZT-FL framework addresses key requirements from the FDA's proposed guidance on AI/ML in medical devices including transparency, robustness and cybersecurity.
- HIPAA Compliance: By implementing end-to-end encryption, secure aggregation and differential privacy, ZT-FL helps ensure compliance with HIPAA's Security Rule.
- GDPR Compliance: The framework's privacy-preserving mechanisms and the principle of data minimization align with GDPR's requirements for data protection and privacy by design.

### 5.10 Practical applicability and generalization

The proposed ZT-FL framework is designed for practical deployment in consumer IoMT environments. This includes smartwatches, home ECG monitors, wearable patches and smartphone-connected healthcare sensors. As the raw patient data remain on local devices, the framework is suitable for scenarios where privacy regulations or institutional policies restrict centralized data collection. However, real-world deployment requires validation on unseen clinical data collected from different populations, sensor types and acquisition conditions. It can be anticipated that the performance may vary when the ECG signal quality is degraded by motion artifacts, poor electrode contact, device differences or demographic shifts between training and deployment populations. From a computational perspective, the proposed CNN architecture is lightweight for many edge-assisted healthcare devices, while more constrained devices may offload parts of the training process to a paired smartphone or local gateway. The additional latency introduced by secure aggregation, encrypted communication and Zero-Trust verification is acceptable for non-emergency monitoring and periodic model updating. However, this may need further optimization for real-time clinical decision support. Therefore, robustness monitoring, periodic recalibration and clinician-in-the-loop validation should be included before deployment in safety-critical healthcare workflows.

### 5.11 Limitations

The present study should be interpreted as a simulation-based proof-of-concept evaluation. First, the experimental validation was performed using a single publicly available ECG dataset, the MIT-BIH Arrhythmia Database, and a simulated federated environment consisting of 20 IoMT clients. Therefore, no physical smartwatch, wearable ECG patch, home-health monitor, or other consumer healthcare device was directly deployed or tested. Second, although the non-IID client configuration was designed to represent heterogeneous IoMT conditions, the generalizability of the proposed framework to different sensor technologies, patient populations, acquisition environments, and real-world signal-quality variations remains to be established through external validation. Third, the security evaluation considered selected attack scenarios, including gradient/model inversion and malicious-update detection, and does not represent the full range of possible adversarial behaviors in operational healthcare networks. Finally, the additional latency associated with secure aggregation, encrypted communication, and Zero-Trust verification is suitable for periodic or non-emergency monitoring but may require further optimization before deployment in real-time emergency or time-critical clinical applications.

Future works will evaluate the framework using external multi-center datasets and real-world wearable-device data to further assess generalization, robustness and clinical reliability.

## 6 Conclusions

This study introduced a novel Zero-Trust Federated Learning (ZT-FL) framework tailored for secure and privacy-preserving artificial intelligence deployment in medical Internet-of-Things (IoMT) systems. The framework integrates three key components: A Zero-Trust Policy Engine (ZTPE) that continuously verifies device integrity and communication trustworthiness, an Adaptive Differential Privacy (A-DP) mechanism that dynamically balances privacy and model utility based on device-specific constraints and secure aggregation protocols that ensure encrypted and tamper-resistant model updates. Together, these components establish a multi-layered defense architecture that addresses the unique privacy, security and compliance requirements of healthcare applications. Experimental results on ECG arrhythmia classification demonstrate that ZT-FL achieves comparable predictive performance to traditional federated learning (92.8% vs. 93.2% accuracy) while offering privacy protection within the stated simulation scope and superior resilience against gradient inversion, model inversion and adversarial attacks. Although the framework introduces modest computational and communication overhead approximately 10% higher latency and 11% higher bandwidth consumption, these trade-offs are acceptable given the substantial improvements in data protection and model robustness. The cost analysis further indicates that while ZT-FL incurs a higher initial investment, its long-term value is realized through reduced risk of data breaches, avoidance of regulatory penalties and enhanced patient safety. Over a five-year period, the total cost of ownership remains justifiable for mission-critical healthcare deployments. Overall, the proposed ZT-FL framework establishes a scalable and regulation-compliant foundation for trustworthy AI in next-generation medical systems. It effectively bridges the gap between federated intelligence and Zero-Trust security thereby aligning with global healthcare standards for clinical safety, patient privacy and ethical AI governance. However, further validation is required using external factors before actual clinical deployment, particularly on unseen patient populations, different wearable sensor platforms and real-world noisy ECG recordings. Future research will focus on improving computational efficiency with hardware-accelerated privacy mechanisms. It will also enable cross-silo collaboration among healthcare institutions and use formal verification to ensure security. Additional directions include blockchain-based audit trails for decentralized trust and federated unlearning for selective data removal. The framework will be validated in real-world clinical settings to advance privacy-centric AI in connected healthcare. By targeting consumer IoMT wearables and companion apps, ZT-FL aims to build trust in everyday health electronics. Future efforts will also emphasize on-device PQC accelerators and AI-guided security testing pipelines for app releases.

## Data Availability

The original contributions presented in the study are included in the article/Supplementary Material, further inquiries can be directed to the corresponding author.
